# Evaluation of cardiovascular risk factors in women referring to health centers in Tabriz, Iran, 2017

**DOI:** 10.15171/hpp.2018.45

**Published:** 2018-10-27

**Authors:** Roghayeh Molani Gol, Maryam Rafraf, Mohammad Asghari Jafarabadi

**Affiliations:** ^1^Students’ Research Committee, Tabriz University of Medical Sciences, Tabriz, Iran; ^2^Nutrition Research Center, Department of Community Nutrition, Tabriz University of Medical Sciences, Tabriz, Iran; ^3^Road Traffic Injury Research Center, Tabriz University of Medical Sciences, Tabriz, Iran; ^4^Department of Statistics and Epidemiology, Tabriz University of Medical Sciences, Tabriz, Iran

**Keywords:** Cardiovascular risk factors, Women, Obesity, Dyslipidemia, Petrochemical region

## Abstract

**Background:** Cardiovascular disease (CVD) is the leading cause of mortality among men and women around the world. The aim of this study was to investigate major cardiovascular risk factors in women living in the Tabriz petrochemical region, Iran during spring 2017.

**Methods:** In this cross-sectional study, a sample of 152 women aged 30-55 years was selected from who attended health center in Tabriz, Iran. Anthropometric measurements, blood pressure,daily dietary intakes and fasting serum lipid profile, oxidized LDL (ox-LDL) and high sensitivity C-reactive protein (hs-CRP) were evaluated.

**Results:** The prevalence of overweight, general and abdominal obesity (based on Body mass index [BMI] and waist circumference [WC]) was 34.2%, 52.6%, and 73.7%, respectively. Eleven point two percent and 4.6% of women had pre-hypertension based on systolic blood pressure (SBP)and diastolic blood pressure (DBP). High serum triglyceride (TG), total cholesterol (TC), low density lipoprotein cholesterol (LDL-C) and low high-density lipoprotein cholesterol (HDL-C)were determined in 32.5%, 25.7%, 17.8% and 56.6% of subjects, respectively. The median of serum ox-LDL concentration was 3181.5 ng/L. Sixty-five point eight percent of participants hadhigh hs-CRP levels. In the multiple-adjusted quintile regression analysis, significant relationships were found between serum ox-LDL and age (B = 96.7, P = 0.003) and between serum hs-CRP with diastolic blood pressure (B = 0.1, P = 0.083) and TG (B = 0.01, P = 0.088).

**Conclusion:** The high prevalence of cardiovascular risk factors in the studied women warrants more public health attention. The results also suggest that aging was associated with high serumox-LDL and increased serum hs-CRP levels, which may reflect enhanced DBP and serum TG.

## Introduction

Health promotion is one of the leading and high-priority dimensions of public health.^1^ Cardiovascular disease (CVD) is one of the most important health problems and the leading cause of mortality among men and women around the world. It is the main reason for disability and most predictable non-communicable diseases.^2,3^ According to the World Health Organization (WHO), there are 17.5 million deaths annually due to CVD in the world and nearly 30% of all deaths are due to these diseases.^4^ CVD mortality rate was reported to be 203 and 331 per 100 000 person-years in women and men of Isfahan, Najafabad and Arak, Iran.^5^ In the study by Azizi et al 78% of men and 80% of women aged ≥30 years in Tehran, Iran presented at least one CVD risk factor.^6^

Risk factors for CVD are classified into the non-modifiable group such as age, inheritance, gender and ethnicity, as well as the modifiable group. The major modifiable risk factors for CVD include obesity, hypertension, dyslipidemia, diabetes mellitus, less physical activity, nutrition, low social economic status and smoking.^2^ Chronic exposure to air pollution is also a proven risk factor for CVD.^7^

Obesity is associated with oxidative stress and a number of cardiovascular risk factors, including hypertension and dyslipidemia.^8^ Among the main risk factors of dyslipidemia, high serum low-density lipoprotein cholesterol (LDL-C) and oxidized LDL (ox-LDL) and decreased high-density lipoprotein cholesterol (HDL-C) concentrations are important factors.^9,10^ Results of a population-based survey in Girona, Spain, showed that obese subjects were at high risk of elevated circulating concentrations of ox-LDL and C-reactive protein (CRP).^11^ Elevated levels of ox-LDL is a proven biomarker in atherosclerotic CVD and plaque formation.^12^ High-sensitivity CRP (hs-CRP) is one of the acute phase proteins and is an indicator of systemic inflammation. Its serum concentration increases in cases of inflammation, infection and coronary artery disease and plays an important role in clinical and prognostic implications in CVD.^12,13^

Epidemiological studies show that people who living in cities with high levels of pollution are at high risk of heart disease.^14,15^ In addition, it has been displayed that women are more at risk for living in the polluted environments, therefore more exposed to environmental hazards.^16^

Different studies have reported that petrochemical industry is one of the main causes and sources of industrial air pollution. Exposure to pollutants released by the petrochemical industry such as particular matter has been found to be a causal factor in oxidative stress-mediated damage to mitochondria and adipose tissue genes and increased pulmonary arterial pressure.^17-19^ Results of a study on Italian gas and energy workers showed that people over 45 years have a high risk of obesity, blood pressure (BP), cholesterol and triglycerides (TG).^20^

The petrochemical industry is an important industry from the viewpoints of employment and economics, in Iran.^17^ However, no published data are available about health status of people living in the petrochemical region, in Iran.

Kojovar is one of the historical areas, located in the southwest of Tabriz, Iran, near the petrochemical and refinery companies with a population of 6000 and 1744 households. Information from the local Environmental Protection Agency shows that the amount of air pollutants is greater than the normal range in this area. Therefore, we initiated a study to evaluate the prevalence of some cardiovascular risk factors including obesity, BP, serum lipid profile, ox-LDL and hs-CRP levels and their relationship in women referring to health center in Kojovar, Tabriz, Iran.

## Materials and Methods

### Study population

In this cross-sectional study, a sample of 152 women (30-55 years old) who attended health center was selected using convenience sampling method in Kojovar during spring (April–May) 2017. The sample size with regard to the prevalence of 39.8% (95% CI: 37.8-41.9) pre-hypertension in Iran,^21^ and considering a 95% confidence level, a power of 80% and tolerable error of 20%, in two-tailed tests using G-Power 3.1.2 software was calculated to be 145 subjects. At total, 160 women were selected. All participants were informed of the study procedure and signed written informed consent. Exclusion criteria were: being pregnant or lactating, menopause, athlete (Individuals with regular and intense physical activity), smoking and women having a history of the disease (such as heart disease, hypertension, diabetes, hepatic, kidney and nervous disease, cancer etc) or with use of medication (for example glucose, lipid and inflammatory lowering drugs) or nutritional supplements (such as vitamin E, zinc and any antioxidants). All of these women were lived for at least 10 years in this area.

### Demographic measures 

General information such as age, educational levels (high school or less and above high school), marital status (single and married), job status (housewife and practitioner), etc was collected by using a questionnaire.

### Anthropometric assessments

Body weight was measured using a calibrated Seca scale and was approximately recorded to 0.5 kg. The participants were measured barefoot wearing light clothing. Height was measured using a mounted tape with the participants’ arms hanging freely at their sides and was approximately recorded to 0.5 cm. Body mass index (BMI) which describes general obesity, was calculated as the weight in kilograms divided by the square of height in meters. BMI categories were defined as underweight (BMI <18.5 kg/m^2^), healthy weight (BMI = 18.5–24.9 kg/m^2^), overweight (BMI = 25.0–29.9 kg/m^2^) and obesity (BMI ≥30 kg/m^2^). Waist circumference (WC) was measured at the level midway between the lowest rib margin and the iliac crest. Measurements were taken with a tape measure in centimeters and rounded to 0.5 cm while the subject was standing. Waist-hip ratio (WHR) is obtained by dividing the size of the waist by the hip circumference. Abdominal obesity was defined as a WC ≥88 cm or WHR ratio ≥0.8.^22^

### BP measurements

BP was measured in the morning by using a mercury sphygmomanometer together with an adult cuff. It was gaged on the upper right arm, which the arm horizontally on a table, and subsequently 5 min rest in the sitting position. Systolic blood pressure (SBP) and diastolic blood pressure (DBP) were measured as the first detectable sound and the disappearance of Korotkoff sounds, respectively. Two readings were recorded at a wait of 1–2 minutes, and the cuff was totally deflated between readings. The mean of the 2 readings was calculated for analysis. Normal BP, pre-hypertension, stage 1 hypertension and stage 2 hypertension were defined as SBP/DBP <120/80 mmHg, SBP/DBP = 120-139/80-89 mm Hg, SBP/DBP = 140-159/90-99 mg/dL and SBP/DBP ≥160/100 mg/dL, respectively.^22^

### Laboratory methods

Venous blood samples (5 mL) were achieved from all participants next a 12-hour overnight fast. Serum was separated by using centrifugation and kept frozen at -70^º^C until assay.

Serum lipid profile measurements were done by using the commercial kits (ParsAzmoon kits, Tehran, Iran). Enzymatic methods were applied by cholesterol esterase, cholesterol oxidase, and glycerol phosphate oxidase for TG and total cholesterol (TC). TG, TC and HDL-C levels were obtained via the autoanalyzer (alcyon 300 automated biochemistry analyser; Abbott laboratories, Abbott Park, IL, USA). When internal quality control met the suitable criteria, all samples were analysed. Cut-off values for TG<150 mg/dL was defined as normal, TG = 150-199 mg/dL as borderline high, TG = 200-499 mg/dL as high and TG ≥500 mg/dL as very high and TC <200 mg/dL is desirable. Serum HDL-C measurement was based on precipitation procedure of the apolipoprotein B-containing lipoprotein whit magnesium chloride fluid and phosphotungstic acid. Inter- and intra-assay coefficients of variation (CV) were 2.0 % and 0.5 % for HDL-C, respectively. HDL-C >50 mg/dL was defined as optimal. LDL-C was later calculated indirectly by the Friedewald formula: LDL-C = TC- (HDL-C+ TG/5). LDL-C <130 mg/dL is normal.^22,23^

Serum ox-LDL levels was measured using ELIZA microplate reader (Awareness, Model stat fax 2000) through sandwich enzyme-linked immune sorbent assay procedure (ELISA kit, bioassay technology laboratory, China) with the murine monoclonal antibody mAB-4E6 as the receive antibody, and a peroxidase conjugated antibody contrary to oxidized apolipoprotein B (Apo B) link to the solid phase (ox-LDL, Mercodia AB, Uppsala, Sweden).^24^ Intra- and inter-assay CV were CV<8% and CV<10%, respectively. Assay range: 40 ng/L–10 000 ng/L.

Hs-CRP was evaluated with an immunoturbidimetric assay by using a biochemistry analyser (alcyon 300) (ParsAzmoon kits, Tehran, Iran). Cut-off values for hs-CRP were categorized as: <1.0 mg/L (low risk), 1.0- 3.0 mg/L (intermediate risk) and ≥ 3.0 mg/L (high risk).^22,25^

### Dietary intakes 

Information about daily vitamin E and vitamin C intakes was obtained by the 24 hours recall questionnaire for 3 days, with 2 weekdays and 1 weekend day, then analysed by Nutritionist 4 software (First Databank Inc., Hearst Corp., San Bruno, CA, USA). Recommended dietary allowances (RDA) for vitamin E, vitamin C and zinc is 15 mg/d, 75 mg/d and 8 mg/d, respectively.^26^ Daily vitamin E, vitamin C and zinc intakes < 75% of RDA were considered as the deficiency.^22^

### Statistical analysis

Statistical analysis was done by STATA software version 13 (Stata Corp, College Station, Texas 77845 USA). Normality of the numeric variables was checked by Kolmogorov-Smirnov test. Data were presented using mean (SD), median (percentile 25-75) and frequency (percent) for the normal, non-normal and categorical variables, respectively. To assess the relationship between serum ox-LDL and hs-CRP levels with studied variables, univariate and multivariate quantile regression modelling was used due to non-normal distribution of the dependent variables. In the multivariate model, the effect of confounders were adjusted and the simultaneous relationship of the predictors were assessed. In all analyses, *P* values less than 0.1 were considered as significant and 90% CI of the regression coefficients were presented.

## Results

In the first stage, 160 women were selected that 8 subjects were lost. The final sample included 152 women. The general and clinical characteristics of the participants are shown in Table 1. The average age of subjects was 40.21 years. Overweight and general obesity were seen in 34.2% and 52.6 % of subjects, respectively. Seventy-three percent of subjects had abdominal obesity based on WC category and 79.4% based on WHR classification. Frequency of subjects with pre-hypertension were 11.2% and 4.6% based on SBP and DBP, respectively.

Biochemical measures of subjects were summarized in Table 2. Abnormal TG, TC, LDL-C and HDL-C levels were identified in 32.5%, 25.7%, 17.8% and 56.6% of subjects, respectively. Median of serum ox-LDL levels was 3181.5 ng/L (2666.2- 4665.5). High hs-CRP levels were determined in 65.8 % of the participants.

Table 3 presents the association between ox-LDL concentration and the other variables. In the multiple-adjusted quantile regression analysis, serum ox-LDL was positively associated with age. No significant associations were found between ox-LDL concentration and other variables (SBP, DBP, serum lipid profile, obesity values and dietary intakes).

Table 4 shows the relationship between serum hs-CRP levels and the studied variables. In the multiple-adjusted quantile regression analysis, serum hs-CRP was positively associated with TG and DBP. There were no significant relationship between hs-CRP levels and TC, LDL-C, HDL-C, anthropometric indexes and dietary intakes data.

## Discussion

There are limited reports about health conditions of Iranian women. The present study is the first one to investigate the cardiovascular risk factor status among women in high air polluted area in Tabriz, Iran.

Obesity is related to various metabolic disorders and augmented cardiovascular mortality.^27^ According to the results, overweight, general obesity, and abdominal obesity were common in the studied subjects. Percentage of obesity in our population was higher than that in other provinces of Iran. In the study by Moghimi-Dehkordi et al overweight and obesity (BMI) was revealed in 36.9% and 20.6%, of females in Tehran, Iran.^28^ In another study by Hajian-Tilaki et al on women living in the north of Iran, rates of overweight, obesity (BMI), and abdominal obesity were 34.8%, 18.8%, and 28.3%, respectively.^29^

Our results regarding obesity values were higher even than those reported for women of some other countries. In the study by Reynolds et al of Chinese women, the rate of overweight and obesity (BMI) was 26.1% and 5.0%, respectively.^30^ According to the National Health and Nutrition Examination Survey (NHANES), the prevalence of general obesity in US females was 34%.^31^

The prevalence of obesity in the regions with high levels of air pollution has been also studied. In the study by Li et al on 12184 women aged 18-74 living in a primarily industrial province of northeast China, the prevalence of overweight and obesity (BMI) were 43.0% and 48.6%. Obesity had positive association with long-term exposure to ambient air pollution.^18^ Three major pathways were hypothesized to this connection. First, air pollution increases oxidative stress and adipose tissue inflammation, decreased glucose utilization in skeletal muscle and hepatic lipid accumulation which is closely tied to obesity and metabolic syndrome. Second, air pollution elevates the risks for various chronic diseases such as heart disease, respiratory disease, and certain types of cancer which in turn affects body weight status. Third, air pollution disturbs regular physical activity and predisposes people toward excess sedentary behavior.^32^

Arterial hypertension is a major growing health problem because of its association with coronary heart disease, cerebrovascular disease, and renal disease.^13,33^ Based on the exclusion criteria, subjects with known hypertension were excluded in this study. However, pre-hypertension was identified in 11.2% and 4.6% of women based on SBP and DBP, respectively. These rates are lower than that reported for females aged ≥30 years (31.4%) in the south of Iran^34^ and women of other countries such as Jamaica (25.0%) and Korea (47.7%).^35,36^

In the present study, the most predominant form of dyslipidemia was low serum HDL-C. High serum TG, TC, and LDL-C were the other forms of dyslipidemia detected in the studied subjects. Similar to our results, in the study by Latifi et al on 1350 women over 20 years of age in Ahvaz, Iran the prevalence of low HDL-C and high TG, TC and LDL-C were 54.7%, 65.1%, 47.5% and 28.4%, respectively.^37^ Khader et al reported that 38.9%, 51.0% and 41.4% of the Jordanian female aged 25 to 85 years had elevated serum TG, TC and LDL-C, respectively. However, percent of subjects with low HDL-C concentrations (27.9 %) was lesser than that of our participants.^38^

Our findings regarding dyslipidemia were higher than rates reported for 2601 women older than 20 years in Turkey (12.6%, 13%, 11.8% and 10.2% based on high TG, TC and LDL-C and low HDL-C levels, respectively).^39^

It was shown that stress and cellular oxidative damage through the formation of free radicals increase LDL oxidation which result in the development of atherosclerosis.^40,41^ The median of serum ox-LDL in the present study was higher than those reported by other studies. Baser et al demonstrated that, the levels of ox-LDL were 1451.30 ng/L in men and women with vitamin D deficiency and 1618 ng/L in controls. In the study by Gungor et al the mean of ox-LDL was 2350 ng/L in women’s with Low cardiovascular risk.^40,42^ It was also positively correlated with age. Kim et al displayed that plasma ox-LDL progressively and significantly increased with age.^43^ Such findings confirms that in aging and age-related metabolic disorders such as dyslipidemia, hyperglycemia, insulin resistance and metabolic syndrome enhancement of oxidative stress and superoxide anion lead to LDL oxidation.^44^

In the general population, elevated hs-CRP levels can independently forecast the risk of all-cause and cardiovascular mortality.^45^ Our data demonstrated that majority of subjects had high hs-CRP concentrations and its levels were positively correlated with DBP and serum TG. Such relationships have also been identified in other studies.^46-48^ Several mechanisms are involved in this regard. CRP decreases the production of nitric oxide by endothelial cells, increases leukocyte adhesion, platelet activation, oxidation, and thrombosis. On the other hand, CRP upregulates the angiotensin type-1 receptor which mediates the action of angiotensin-II and leads to increased BP.^49,50^ Arterial stiffening was also associated with many circulating inflammatory markers. All these facts indicate that CPR has a role in the development of hypertension.^13,51-53^

The association between TG and hs-CRP levels suggests that TG-rich remnants of chylomicron and VLDL metabolism can penetrate the subintimal space and the arterial wall where they are ingested by macrophages and become proatherogenic foam cells. When the mass of circulating TG increases, the average LDL particle size decreases, and these LDL particles are more susceptible to oxidation, a procedure that increases their atherogenicity.^54^

Overall, diet is a significant determinant of CVD.^41^ Vitamin C and vitamin E are the most important antioxidants and have a protective role in the development of atherosclerotic heart disease by inhibiting LDL oxidation in human plasma.^55,56^ Zinc is an essential nutrient for human health and has anti-inflammatory and anti-oxidative stress functions. Zinc deficiency is also associated with the development of CVD, especially atherosclerosis.^57^ Based on the obtained results, dietary deficiency of these vitamins and zinc was seen in the studied women.

The present study had some limitations. Because of the cross-sectional design of our study, lack of a control group and low sample size, detected results cannot be attributed to the exposure to air pollutants. Longitudinal studies are needed to evaluate the CVD risk factors in people who are exposed to a higher level of air pollution compared with the general population. Raising the awareness of health care providers about the cardiovascular risk factor management, advanced therapies, and educating women about health status are suggested to elevate women’s wellness. The results of current study may be helpful regarding implication for policy and practice and provides a certain baseline for further studies.

## Conclusion

Our study indicated that the cardiovascular risk factors including obesity, pre-hypertension, dyslipidemia, and high hs-CRP were common in studied women. Ox-LDL level correlated with age and hs-CRP with DBP and TG level. More public health attention and supporting primary preventive approaches are needed to modify CVD risk factors and health promotion of women who live in highly polluted regions.

## Ethical approval

The ethical committee of Tabriz University of Medical Sciences approved the study protocol (Ethical code: IR. TBZMED.REC.1396.8). Written informed consent was obtained from all subjects.

## Competing interests

The authors declare no conflicts of interest, both financial and non-financial, for this study.

## Funding

The present study was supported by the Research Vice-chancellor of Tabriz University of Medical Sciences, Tabriz, Iran. The Vice-chancellor had no role in the design, analysis or writing of this article.

## Authors’ contributions

The whole project was designed and conducted by MR, as a supervisor; and RMG contributed to data gathering and on-site study management. Sampling methodology and statistical analysis were performed under the direction of MAJ. All authors contributed to manuscript preparation and approved the final manuscript.

## Acknowledgments

The authors thank all the health care providers and women who participated in the study. This article was written based on data for a MSc. thesis (No: T/A/123), which was registered at the Tabriz University of Medical Sciences.

**Table 1:** General and clinical characteristics in women, Kojovar, Tabriz, Iran, spring 2017

	**Mean or n**	**SD or %**
Age (y)	40.21	5.88
Marital status (%)		
Single	1	0.7
Married	151	99.3
Educational level (%)		
High school or less	116	72.5
Above High school	44	27
Job status (%)		
Housewife	144	94.7
Practitioner	8	5.3
Weight (kg)	76	13.59
BMI (kg/m^2^) (%)	30.48	5.05
<18.5	1	0.7
18.5-24.9	19	12.5
25.0- 29.9	52	34.2
≥30	80	52.6
WC (cm) (%)	94.24	10.87
<88 (cm)	40	26.3
≥88 (cm)	112	73.7
WHR (%)	0.84	0.06
<0.8	33	20.6
≥0.8	127	79.4
SBP (mm Hg)	113.30	9.27
DBP (mm Hg)	71.41	8.08
Normal BP, (mm Hg) (%)		
SBP <120	134	88.2
DBP <80	144	94.7
Pre-hypertension, (mm Hg) (%)		
SBP= 120-139	17	11.2
DBP= 80-89	7	4.6
Hypertension stage 1, (mm Hg) (%)		
SBP= 140-159	1	0.7
DBP= 90-99	1	0.7
Hypertension stage 2, (mm Hg) (%)		
SBP≥160	0	0
DBP≥100	0	0
Vitamin C (mg/d) (%)	72.68	53.94
<75% of RDA	71	46.7
≥75% of RDA	81	53.3
Vitamin E (mg/d) (%)	2.16	1.84
<75% of RDA	151	99.3
≥75% of RDA	1	0.7
Zinc (mg/d) (%)	4.07	2.90
<75% of RDA	134	88.2
≥75% of RDA	18	11.8

Abbreviations: BMI, body mass index; WC, waist circumference; WHR, waist-hip ratio; BP, blood pressure; SBP, systolic blood pressure; DBP, diastolic blood pressure.

**Table 2:** Biochemical parameters in women, Kojovar, Tabriz, Iran, spring 2017

	**Mean or n**	**SD or %**
TG (mg/dL) (%)	124.55	69.47
<150	108	67.5
150-199	27	16.9
200-499	17	10.6
≥500	8	5.0
TC (mg/dL) (%)	177.73	32.67
<200	113	74.3
≥200	39	25.7
HDL-C (mg/dL) (%)	50.52	14.54
≤50	86	56.6
>50	66	43.4
LDL-C (mg/dL) (%)	102.29	28.96
<130	125	82.2
≥130	27	17.8
Ox-LDL (ng/L)^a^	3181.5 (2666.2-4666.5)
Hs-CRP (mg/L)^a^	4.05 (2.50-6.90)
<1.0 (mg/L) (%)	2	1.3
(1.0-3.0) (mg/L) (%)	50	32.9
≥3.0	100	65.8

Abbreviations: TG, triglyceride; TC, total cholesterol; HDL-C, high-density lipoprotein cholesterol; LDL-C, low-density lipoprotein cholesterol; ox-LDL, oxidized LDL; hs-CRP, high-sensitivity C- reactive protein ^a^Median (percentile 25-75)

**Table 3:** Associations between circulating ox-LDL (ng/L) concentrations and studied variables in women, Kojovar, Tabriz, Iran, spring 2017

	**Unadjusted**		**Adjusted**	
	**B (90% CI) **	***P*** ** value**	**B (90% CI)**	***P*** ** value**
Age (y)	92.0 (43.0 to 140.9)	0.002	96.7 (42.9 to 150.6)	0.003^a^
Weight (kg)	-0.6 (-22.5 to 21.3)	0.963	-	-
BMI (kg/m^2^)	-16.6 (-71.7 to 38.4)	0.617	-76.5 (-222.1 to -69.0)	0.386
WC (cm)	-1.0 (-27.9 to 25.8)	0.949	26.6 (-58.7 to 112.0)	0.607
WHR	4036.5 (-484.9 to 8558.0)	0.142	888.3 (-7209.1 to 8985.9)	0.856
SBP (mm Hg)	4 (-26.3 to 34.3)	0.828	-18.7 (-67.9 to 30.4)	0.529
DBP (mm Hg)	4 (-31.4 to 39.4)	0.852	-3.8 (-59.5 to 51.8)	0.909
TG (mg/dL)	2.3 (-2.0 to 6.7)	0.371	1.4 (-3.0 to 6.0)	0.590
TC (mg/dL)	8.4 (-0.7 to 17.5)	0.129	7.5 (-1.9 to 17.0)	0.190
LDL-C (mg/dL)	7.4 (-2.0 to 16.9)	0.198	-	-
HDL-C (mg/dL)	24.4 (3.1 to 45.6)	0.059	9.3 (-10.5 to 29.3)	0.436
Vitamin C^b^ (mg/d)	-1.8 (-7.3 to 3.5)	0.573	-5.2 (-11.1 to 0.6)	0.142
Vitamin E^b^ (mg/d)	56.7 (-100.4 to 213.8)	0.551	19.3 (-141.8 to 180.5)	0.843
Zinc^b^ (mg/d)	1.1 (-94.2 to 96.4)	0.985	5.3 (-170.0 to 180.7)	0.960

Abbreviations: BMI, body mass index; WC, waist circumference; WHR, waist-hip ratio; SBP, systolic blood pressure; DBP, diastolic blood pressure; TG, triglyceride; TC, total cholesterol; LDL-C, low-density lipoprotein cholesterol; HDL-C, high-density lipoprotein cholesterol; ox-LDL, oxidized LDL. *P* value based on univariate and multivariate quantile regression. ^a^ *P* < 0.1 is significant. ^b^ Daily intake.

**Table 4:** Associations between circulating hs-CRP (mg/L) concentrations and studied variables in women, Kojovar, Tabriz, Iran, spring 2017

	**Unadjusted**		**Adjusted**	
	**B (90% CI) **	***P*** ** value**	**B (90% CI)**	***P*** ** value**
Age (y)	0.0 (-0.0 to 0.1)	0.630	-0.0 (-0.1 to 0.1)	0.800
Weight (kg)	0.0 (-0.0 to 0.0)	0.105	-	-
BMI (kg/m^2^)	0.0 (-0.0 to 0.2)	0.222	0.0 (-0.4 to 0.4)	0.991
WC (cm)	0.0 (0.0 to 0.1)	0.029	0.0 (-0.1 to 0.2)	0.687
WHR	6.8 (-0.7 to 14.3)	0.136	-9.4 (-32.1 to 12.8)	0.479
SBP (mm Hg)	0.0 (-0.0 to 0.1)	0.102	-0.0 (-0.1 to 0.0)	0.461
DBP (mm Hg)	0.0 (-0.0 to 0.1)	0.184	0.1 (0.0 to 0.3)	0.083^a^
TG (mg/dL)	0.0 (-0.0 to 0.01)	0.174	0.0 (0.0 to 0.0)	0.088^a^
TC (mg/dL)	0.0 (-0.0 to 0.0)	0.216	0.0 (-0.0 to 0.0)	0.245
LDL-C (mg/dL)	0.0 (-0.0 to 0.0)	0.668	-	-
HDL-C (mg/dL)	-0.0 (-0.0 to 0.0)	0.900	0.0 (-0.0 to 0.1)	0.150
Vitamin C^b^	-0.0 (-0.0 to 0.0)	0.731	-0.0 (-0.0 to 0.0)	0.792
Vitamin E^b^	-0.0 (-0.3 to 0.3)	0.936	0.0 (-0.4 to 0.4)	0.952
Zinc^b^ (mg/d)	-0.0 (-0.2 to 0.1)	0.884	-0.3 (-0.8 to 0.1)	0.232

Abbreviations: BMI, body mass index; WC, waist circumference; WHR, waist-hip ratio; SBP, systolic blood pressure; DBP, diastolic blood pressure; TG, triglyceride; TC, total cholesterol; LDL-C, low-density lipoprotein cholesterol; HDL-C, high-density lipoprotein cholesterol; ox-LDL, oxidized LDL. *P* value based on univariate and multivariate quantile regression. ^a^ *P* < 0.1 is significant. ^b^ Daily intake.
